# Wheat Fusarium Head Blight Automatic Non-Destructive Detection Based on Multi-Scale Imaging: A Technical Perspective

**DOI:** 10.3390/plants13131722

**Published:** 2024-06-21

**Authors:** Guoqing Feng, Ying Gu, Cheng Wang, Yanan Zhou, Shuo Huang, Bin Luo

**Affiliations:** 1 Research Center of Intelligent Equipment, Beijing Academy of Agriculture and Forestry Sciences, Beijing 100089, China; grigorii_f@yeah.net (G.F.); yinggu17@yeah.net (Y.G.); wangc@nercita.org.cn (C.W.); zhouyn@nercita.org.cn (Y.Z.); huangs@nercita.org.cn (S.H.); 2 National Engineering Research Center for Information Technology in Agriculture, Beijing 100097, China; 3 College of Agricultural Engineering, Jiangsu University, Zhenjiang 212000, China

**Keywords:** wheat FHB, phenotyping, imaging technique, advanced technology

## Abstract

Fusarium head blight (FHB) is a major threat to global wheat production. Recent reviews of wheat FHB focused on pathology or comprehensive prevention and lacked a summary of advanced detection techniques. Unlike traditional detection and management methods, wheat FHB detection based on various imaging technologies has the obvious advantages of a high degree of automation and efficiency. With the rapid development of computer vision and deep learning technology, the number of related research has grown explosively in recent years. This review begins with an overview of wheat FHB epidemic mechanisms and changes in the characteristics of infected wheat. On this basis, the imaging scales are divided into microscopic, medium, submacroscopic, and macroscopic scales. Then, we outline the recent relevant articles, algorithms, and methodologies about wheat FHB from disease detection to qualitative analysis and summarize the potential difficulties in the practicalization of the corresponding technology. This paper could provide researchers with more targeted technical support and breakthrough directions. Additionally, this paper provides an overview of the ideal application mode of the FHB detection technologies based on multi-scale imaging and then examines the development trend of the all-scale detection system, which paved the way for the fusion of non-destructive detection technologies of wheat FHB based on multi-scale imaging.

## 1. Introduction

Wheat, one of the world’s three largest food crops, covers the main food needs of more than 3 billion people [1] and provides the global population with essential amino acids, minerals, and other vital trace elements [2]. The statistics of the Food and Agriculture Organization of the United Nations (FAO) show that, over the past 15 years, wheat-seeded areas have consistently ranked first among 158 primary crops, and the global wheat production in 2023 was approximately 787 million tons. Wheat production safety is paramount, whether in global food security [3], economic impact, or future crop strategies [4].

Over the years, FHB caused by various fungi such as *Fusarium graminearum* has become a globally important fungal disease affecting wheat [5], second only to leaf rust in terms of damage. According to statistics, the loss of wheat production caused by wheat FHB has reached more than 1% worldwide, 3.20% in the Midwestern United States and Canada, and 8.75% in Mainland China [6]. In terms of economic benefits, the widespread occurrence of FHB has resulted in significant economic losses [7]. From 1998 to 2000, the direct and indirect economic losses caused by FHB on major food crops amounted to approximately $2.67 billion [8]. From the perspective of environmental impact, the absence of information on the severity of pathogenic fungi or plant infections is highly likely to lead to the misuse of chemical pesticides, resulting in environmental pollution and reduced biodiversity [9]. The increase in economic risks and the deterioration of land resource quality will inevitably reduce the production enthusiasm of farmers and the sustainability of agricultural production development. Moreover, the toxin produced by FHB, mainly deoxynivalenol (DON), poses a significant threat to human and animal health [10]. Therefore, early and accurate detection, monitoring, and assessment of this disease are crucial for farmers, managers, and decision-makers.

Field surveys, as the most primitive and basic method of disease research, require relevant personnel to have lots of expertise in plant pathology and epithetics. Biochemical technology, as another mainstream technique, requires more sophisticated sampling and processing and can cause irreversible damage to experimental targets [11,12]. Both methods commonly used in the late stages of infection are practically effective [13]. However, such labor-intensive methods are costly and time-consuming, limiting not only applications in large-scale detection scenarios but also easily missing the best time to use antimicrobials to prevent disease. The prevalence of FHB is widespread, and even with the support of genomic tools, genetic engineering-mediated FHB-resistant genotype breeding progresses slowly [14]. Therefore, the demand for accurate early detection of wheat FHB or large-scale, real-time, and non-destructive testing during its high incidence period is becoming increasingly prominent.

Over the past few decades, with the rapid development of computer science, bioinformatics, and other disciplines, massive techniques of fast, accurate, low-cost, automatic, non-destructive detection of wheat diseases have emerged. Researchers have been able to achieve tasks related to the detection, segmentation, grading, and counting of wheat FHB using a range of techniques, from digital image processing techniques that can quickly process images to traditional machine learning, which is suitable for structured data and feature engineering, to deep learning that excels in handling unstructured data and complex tasks, with the updates and iterations of technology [15,16,17]. However, from previous research on wheat FHB detection, we can find that although digital image processing technology has made significant breakthroughs compared to visual detection, the lack of accuracy and spatiotemporal generalization ability is still a major problem [18]. Although traditional machine learning requires more complex manual design, such methods are more focused on the pathogenesis and characteristics of wheat and Fusarium head blight and are, therefore, widely used [19]. The strong generalization ability, stability, and accuracy of deep learning technology in this detection task are self-evident, but the problem of high data demand and shortage cannot be ignored [20].

It is the constantly evolving imaging technology that provides adequate and valuable data for these methods. With the support of various imaging technologies, detection methods typically have characteristics such as high information richness, real-time performance, non-destructive, automation, and efficiency [21]. Taking the detection techniques formed by the most commonly used RGB imaging and spectral imaging as examples, compared to traditional detection techniques, the former analyzes the target object by obtaining important information such as color features and texture details, which has the characteristics of more strong intuitiveness and simplicity; The latter is known for reflecting the chemical composition, material characteristics, and spectral characteristics of objects, and has more powerful feature resolution and classification ability for different substances.

Barbedo [22] discussed the challenge of plant disease detection by utilizing leaf disease detection as an example. However, they only reviewed the detection within the research about visible light, which would ignore massive other valuable information. The cutting-edge techniques used for detecting wheat rust, which mainly attacks the leaves and stems of wheat, were comprehensively discussed in [23]. They discussed the challenges and limitations associated with remote sensing, machine learning (ML), deep learning, and Internet of Things (IoT) technologies in this field. While wheat FHB can cause spike disease that has the greatest impact on safe production, it has not been effectively summarized and given a more forward-looking perspective at the technical level of automatic detection without damage. This is one of the reasons why we wrote this review. 

**Figure 1:**
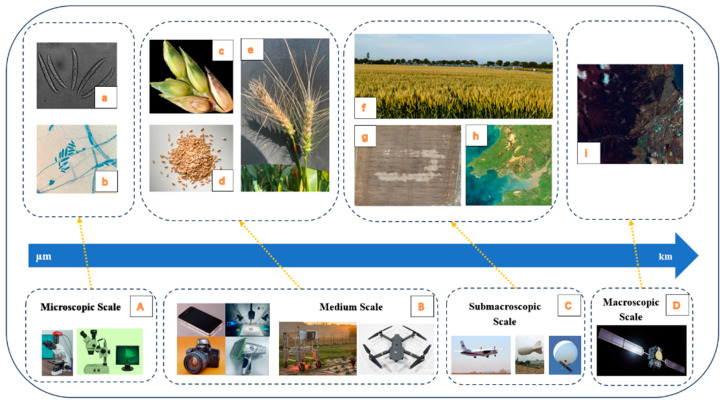
Common platforms and imaging in wheat FHB detection. (**A**) Microscope equipped with an industrial camera and scanning electron microscope; (**B**) smartphone, indoor spectral measurement platform, ordinary camera, handheld spectrometer, ground-based platform, and light UAV; (**C**) Remote sensing aircraft, airship, and balloon; (**D**) satellite; (**a**,**b**) fusarium spores; (**c**–**e**) kernel on the ear, scattered kernel and single spike; (**f**–**h**) wheat field captured at heights of 0.5 m and 30 m, and remote sensing images; (**i**) satellite images.

As shown in Figure 1, with the rapid development of imaging platforms such as microscopes [24], drones [25], and remote sensing satellites [26], automatic wheat FHB detection has obtained more multi-scale images with rich experimental and application value. According to the differences between each imaging scale, this paper divided them into ‘microscopic scale’ (scale range is very small, such as cells, molecules, etc.), ‘medium scale’ (scale range is between microscopic and submacroscopic scale, such as crop organs, single grain, individual plant, etc.), ‘submacroscopic scale’ (scale range can be from several meters to thousands of meters, such as experimental fields, large planting areas, etc.), and ‘macroscopic scale’ (largest scale range, such as ecosystem, earth, etc.), in order to clarify the technical scope for subsequent discussions.

A few excellent advanced studies from every imaging scale are displayed in Table 1. These mostly have been proposed in the past 5–8 years, which is consistent with the outbreak time point of the number of research papers on wheat FHB [27]. During this period, thanks to the rapid development of spectral imaging technology, deep learning algorithms, and other technological elements, automatic non-destructive detection of wheat FHB has also received significant attention. Within these contexts, this review was based on various mainstream imaging technologies, mainly including but not limited to RGB and spectral imaging, and reviewed the advanced automatic non-destructive wheat FHB detection technologies. The research articles reviewed were based on non-biochemical techniques for wheat FHB detection since 2018, which were searched in Google Scholar utilizing the keyword ‘wheat FHB detection’. A total of 62 articles were obtained, and after sorting the research objectives, they were classified into the four imaging scales mentioned above. Firstly, this article briefly provides an overview of the infection factors and transmission routes of wheat FHB and explains the changes in physical and physiological biochemical wheat characteristics after infection. Secondly, based on the proposed four imaging scales, the research achievements and challenges in the past few years are discussed according to the main research directions of recent studies. We mainly summarize the relevant articles, algorithms, and methods from disease detection to qualitative analysis and summarize some potential difficulties in the practicalization of technology. Finally, the future development trend of automatic non-destructive detection for wheat FHB is discussed, and prospects are made from a technical perspective.

## 2. The Epidemic of FHB and Its Impact on Wheat Characteristics

### 2.1. Infection and Broadcasting of Wheat FHB

Under realistic conditions, the main source of wheat FHB infection is the ascospores of various residues from the previous crop season. According to incomplete statistics, at least 17 common species of causal organizations, including *Fusarium graminearum* Schw, *Fusarium graminearum* (*Gibberella zeae*), *F. culmorum*, *F. avenaceum* (*G. avenacea*), *F. poae*, and *Microdochium nivale* (*Monographella nivalis*), have been associated with the disease [43]. *F. graminearum* is the predominant FHB pathogen in much of the world, especially in the temperate and warmer regions, while *F. culmorum* is more frequently found in the cooler regions of the world [44]. The flowering stage of wheat is the most susceptible stage to FHB, especially within 20 days after full heading. In favorable weather conditions, the inoculum is blown by the wind or splashed by rain and lands on open spikes [11]. Spores germinate on the spikelet to produce mycotoxins. After the stage of pathogenicity, mycotoxins accumulate in the spike-like tissue and grains and infect the spikelet. Then, symptoms gradually appear at the filling stage and eventually break out into FHB disasters at the ripening stage.

In terms of natural factors, suitable temperature, rainfall, and relative humidity around wheat are the main reasons for the proliferation of FHB pathogens [45]. Taking temperature as an example, under normal circumstances, there is little or no infection with FHB below 15 °C. As the temperature increases from 20 °C to 30 °C, the likelihood of wheat being infected with FHB will significantly increase [46]. Additionally, previous studies have shown that with the significant impact of future climate conditions on temperature and humidity, excessive rainfall or drought caused by this will become the largest environmental factor affecting the development of wheat FHB [47]. In addition to natural factors, the influence of some human factors cannot be ignored. For example, late sowing can directly delay or shorten the growth period, returning straws to the field can increase the survival rate of *Fusarium graminearum* due to a large number of crop residues and rich residues, continuous cropping and regular tillage can disrupt soil nutrient balance, reduce soil organic matter, and the use of insecticides without targeting them may not be able to suppress the population dynamics of Fusarium, these operations all will make wheat more susceptible to FHB [48,49,50,51].

**Figure 2:**
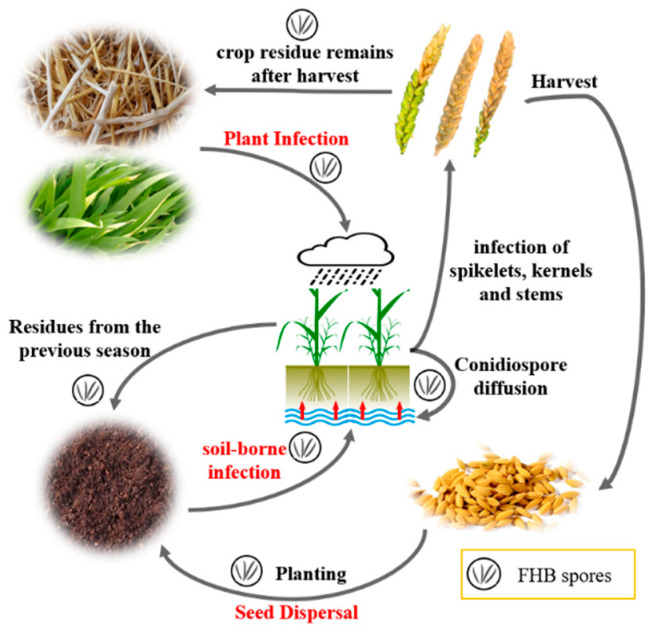
Schematic diagram of FHB propagation and cycle.

As shown in Figure 2, from the perspective of disease transmission, the cycle types of wheat FHB mainly include plant infection, seed dispersal, and soil-borne infection, with the first two methods being the main transmission routes. The main manifestation of plant infection is that pathogens overwinter on residues such as wheat straw and leaves. With the promotion of spring rainfall and the rise of the water table, the pathogen infects plants and continues to spread until before and after harvest. Seed dispersal mainly refers to the parasitism of pathogens in wheat grains, which are preserved until the next year after harvest. The seeds carrying pathogens will cause an epidemic of diseases in the new planting season. The infection rate of seed is also important for the prevalence of FHB. Although the soil-borne infection has little impact, pathogens overwinter in the soil and invade wheat roots or aboveground parts through factors such as roots or rain, ultimately entering the disease cycle.

### 2.2. Changes in Wheat Characteristics Caused by FHB

Wheat infected with FHB exhibits different characteristic changes at each growth stage. Physical features are generally obtained by observing RGB images, mainly manifested as changes in the color, shape, texture, and position of wheat or disease spots. Taking spike rot, which is the most harmful, as an example, in the early stage of disease occurrence, small light brown watery lesions appear on the spikelets and glumes, then gradually expand to the entire spikelet and cause it to wither and turn it yellow. When humidity is high, a pink colloidal mold layer is formed at the lesion [52]. At the later stage, small black spots (perithecium) densely grow on spikes and extend to their axis. After the diseased area withers and browns, the spikelet above the affected area will form chalky white spikes.

Physiological and biochemical characteristics are generally obtained through processing and analysis of spectral images, mainly reflected in changes in chemical substances and tissue structure in various organs of crops. Due to the influence of DON, the main component of vomitoxin from Fusarium, the contents or properties of organic compounds such as proteins, carbohydrates, and lipids in wheat will undergo massive changes [53]. The loss of water and chlorophyll is more significant when the grains or the whole spike are severely infected [54]. 

Chemical substances in crops, including carotenoids, nitrogen, and cellulose, also undergo varying degrees of quantitative changes [55]. In addition, the tissue structures of wheat organs that are altered or damaged by FHB have also been validated in multiple studies [56,57]. As the above-mentioned substances and structures are disrupted, the optical properties of infected and healthy wheat ears show significant differences [58]. This is largely due to photochemical damage caused by the limitation of photoprotective mechanisms such as the chlorophyll cycle and pigment screening. For example, the spectral reflectance of the infected wheat canopy increases in the visible light range and significantly decreases in the near-infrared range [59]. The decrease in chlorophyll content in wheat cells reduces the possibility of photon re-emission and re-absorption within this wavelength range, leading to an increase in spectral reflectance in the chlorophyll band [60]; The reduction of carotenoids will make it more difficult to capture changes in pigment deposition within wheat spikes [61]. By utilizing the changes in optical properties caused by Fusarium, optical detection methods have been widely applied in the non-destructive detection of wheat FHB.

## 3. Detection of Wheat FHB at Microscopic Scale

### 3.1. Overview of Previous Research

Early detection and monitoring of wheat FHB fungus can provide agronomists with information on the concentration and quantity of pathogenic fungus and help them accurately predict the severity of the disease and implement necessary control measures [62]. Before the rapid development of artificial intelligence (AI), Polymerase Chain Reaction (PCR) and Enzyme-Linked Immunosorbent Assay (ELISA) were some of the common chemical methods to detect fungal spores [56,57]. These methods require well-trained personnel to operate using specific laboratory equipment and can cause irreversible damage to the target crops. Moreover, detection under the microscopes, especially considering the scattered and numerous observation points, may require lots of time and manpower. The emergence of traditional ML methods has helped researchers utilize computers instead of manual labor to complete complex observation tasks and gradually achieve non-destructive detection through computer vision technology. These methods typically extract spore microscopic image features and process them to detect targets. With their development and assistance, problems such as unclear spore edge contours caused by inconsistent lighting and uneven background brightness when segmentation spores have been gradually improved [63]. However, traditional ML methods are only suitable for situations with a single target, obvious features, and a simple background [64]. There remain many other funguses in the air that appear as interferences, and detecting the specific type of spores solely through general feature extraction is very challenging.

Deep learning does not require a large amount of feature engineering and can bring models stronger generalization ability. Compared to detection targets at other scales, spores have fewer available features, but the detection spores requires high positioning accuracy. This places higher demands on the model’s ability to extract tiny features and semantic information. Zhang et al. [24] integrated the attention mechanism module and adaptive feature fusion mechanism into the feature pyramid structure of Yolo, tackling issues related to small size, limited characteristics, and unclear attributes of FHB spores. This study improved the average recognition accuracy of *F. graminearum* spores to 98.57%. Meanwhile, the spore images collected by microscopy have trouble with uneven brightness, low background contrast, and spore adhesion. Zhang et al. [28] segmented pixels of FHB spores in challenging circumstances, such as when they contain independent and inter-adherent spores. This method has demonstrated excellent computer vision processing capabilities in tackling issues related to false and missed detection of attached spore images, and rough contours, increasing the overall segmentation and integration ratio of wheat FHB spore to 94.3%. Furthermore, Yuan et al. [65] proposed an effective lightweight model and simulated the realistic environment with various fungal specifications in the field by mixing five common fungal spores in the dataset. The average accuracy of this method reached 88%, while the inference time of the model was only 4.6 ms. Combining the low-cost spore traps proposed by [66] for wheat FHB, it can be affirmed that the practicalization of real-time dynamic detection of wheat FHB spores is ushering in new opportunities.

### 3.2. Challenge

The automatic detection of wheat FHB at the microscopic scale mainly revolves around the detection of Fusarium spores. The existing methods are based on data collected in the laboratory and have achieved certain breakthroughs in model accuracy and practicality. The emergence of low-cost spore traps has greatly reduced reliance on traditional spore-trapping techniques [67]. However, there is currently no research combining field equipment with automatic detection of wheat FHB, which makes it impossible for us to determine the exact reliability of existing methods. In future research, the complexity and diversity of microbial populations in natural farmland, different climate and lighting conditions, morphological changes of FHB spores at different stages, and differences between data acquisition devices are all obstacles to the practicalization of existing work results.

## 4. Detection of Wheat FHB at Medium Scale

### 4.1. Overview of Previous Research on Kernels

Detection of Fusarium Damaged Kernels (FDKs) is essential to early detection and containment of wheat FHB transmission. Wheat kernels infected with FHB often exhibit symptoms such as light weight, chalky white, and atrophy [68]. Diseased seeds can directly or indirectly pollute food and reduce its quality, increasing subsequent costs of cleaning and marketing. Detection of FDKs can ensure reasonable chemical control [69], guide agricultural practices, and assist in screening out FHB-resistant wheat varieties [14]. 

**Figure 3:**
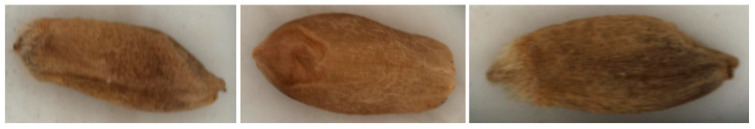
From left to right is a FDK, a healthy kernel, and an unhealthy but non-FDK.

With the assistance of various deep learning methods, the binary classification accuracy of healthy seeds and FDKs based on RGB images has reached 97%. The identification accuracy of FDKs can even reach 99% [70]. However, the challenges in practical application are far more than that. During harvesting, transportation, and storage, excess heat [71] can cause germ or heat-damaged kernels with distorted colors, fungal pathogens [72] can cause discoloration and light damage, and insects [73] usually chew the grains to destroy them. Therefore, such damaged seeds are easily confused with the diseased, which can directly mislead breeding personnel in the quality evaluation and pathogenic factor analysis of the batches of seeds. The difficulty of manual annotation and the lack of large-scale datasets seriously hinder the differentiation between damaged seeds and diseased seeds. WheatSeedBelt [74], a high-resolution large-scale dataset consisting of 40,420 single kernel images of 268 wheat varieties at close-up top and side view, with annotations for kernel status, greatly alleviated this problem. Based on this dataset, relevant researchers established a semi-supervised three-classification model for healthy kernels, unhealthy but non-FDK, and FDKs shown in Figure 3. Among them, all damaged seeds without FHB will rely on extremely meticulous visual judgment and be classified as unhealthy but non-FDK. Although the established model only obtained 68.30% of the f1 score in the three-classification task, compared with the high inconsistency of the prediction results of three human experts, this model is far higher in terms of cost savings and reliability.

Compared to RGB imaging, spectral imaging excellently performs tasks that are difficult to distinguish through external features. In traditional chemical methods, the presence of FDKs is often determined by measuring the DON content in wheat kernels, and many advanced technologies have utilized the method for reference. The analysis is based on hyperspectral data, ref. [75] first identified the absorption bands of DON at 1414 and 1906 nm and proposed that the characteristic bands of FDK are located near 1195, 1208, 1365, 1425, 1440, 1700, 1905, and 2001 nm. Subsequently, Delwiche et al. [76] reduced the number of characteristic bands to four (1100, 1197, 1308, and 1394 nm). However, Nadimi et al. [68] found that the presence of FDKs does not necessarily confirm the presence of DON, meaning that DON cannot serve as the single basis for detecting FDKs. Nevertheless, Liang et al. [77] identified 430–600 nm, which mainly reflected the color difference of wheat grains, as the optimal wavelength and drew one notable conclusion that the visible near-infrared (400–1000 nm) region was more accurate than the short-wave infrared (1000–2500 nm) region. On the contrary, Almoujahed et al. [78] observed that wheat varieties with high resistance to FHB had lower reflectance in 400–1000 nm, while reflectance was higher in 1000–1650 nm. This indicated that wheat kernels with different resistance to FHB may even extract opposite features. In addition to feature extraction with hyperspectral imaging, Raman spectroscopy can provide molecular-level information while compensating for the issue of unclear absorbance of infrared spectra. As a method beneficial for biological sample analysis [79], Raman spectroscopy has also achieved an accuracy of 93.62% in more refined FDK severity grading with the combination of machine learning [80].

In spectral research closer to the application side, as an important part in FDK automatic detection, the addition of a conveying device enhances the automation level of the entire process flow. Barbedo et al. [81] developed a set of algorithms based on the simple transport device that has strong robustness to factors such as shape, direction, shadows, and clustering of the kernels. The improvement of automation efficiency requires probing the optimal ratio of transmission speed to imaging quality or improving throughput in one detection. Delwiche et al. [76] increased the number of kernels once a scanning, from 50 in previous HSI studies to over 200, reducing processing time to the millisecond level per kernel.

### 4.2. Challenges with Kernel Detection

The methods for obtaining ground truth in recent FDK detection research can be roughly divided into visual evaluation and DON toxin qualitative detection. Najafian et al. [74] validated the low consistency of human expert ratings based on utilizing their large-scale dataset. Such low consistency directly affects the knowledge and final effectiveness of model learning. However, the existing qualitative standards for DON toxins only define a single threshold for DON concentration (1000 ppb), which is insufficient to guide researchers in subdividing the infection level of seed. This also leads to most current spectral-based FDK detection merely performing binary classification. How to subdivide classification criteria to assist breeding work is the next issue that remains to be tackled. Furthermore, considering the complexity of seed storage environments, compared to the subdivision of infection levels, the three-classification task of health kernels, unhealthy but non-FDK, and FDKs is more closely related to the application requirements of large-scale detection of damaged and diseased seeds in production. There remains a lack of in-depth research based on spectral technology for this task.

### 4.3. Overview of Previous Research on Ears

Due to the convenience of obtaining data at a medium scale and the rapid development of various imaging and computer vision technologies, most FHB detection studies have focused on the wheat ear level. In the past 5 years alone, imaging techniques applied to FHB detection at the wheat ear level included but were not limited to RGB imaging, hyperspectral imaging (HSI), multispectral imaging (MSI), infrared thermal imaging (IRT), hyperspectral microscopy (HMI), chlorophyll fluorescence imaging (CFI), and high-throughput phenotype (HTP). Therefore, this section will review both RGB and spectral imaging techniques.

#### 4.3.1. Detection Based on RGB Imaging

Profit from the rapid development of mobile smart devices, low-cost RGB imaging technology has been applied in more small-scale detection scenarios. For researchers, easily accessible imaging equipment and simple imaging principles greatly reduce the cost of large-scale data acquisition and analysis. For agricultural producers and operators, they also make wheat FHB detection on based RGB imaging at the medium scale the easiest to obtain and master.

**Data acquisition:** As the imaging scale with the longest development time and the greatest influence, at the medium scale, many excellent image datasets such as PlantVillage [82] have been proposed. These datasets still serve as the mainstay in many studies, but most of them are taken indoors. With further research, Mohanty et al. [83,84] pointed out that models based solely on laboratory data cannot withstand inspection of detection in wheat fields. However, with the peak of research on the automatic detection of wheat FHB, more pluralistic data have started their applications: multiple varieties [85], omnidirectional [86], multiple weather conditions and backgrounds [87], multiple raters and years [88], multiple growth stages and image resolutions [89], etc.

**Data processing:** The importance of robustness and generalization ability of methods becomes more prominent when the data dimensions increase. Zhang et al. [90] combined color features extracted from HSV and CMYK color spaces with high-dimensional features extracted from ResNet as comprehensive features of wheat ears. The model obtained by this fusion has excellent spatiotemporal generalization ability and has been validated on wheat fields in different years and regions. On the basis of similar feature fusion methods, Gu et al. [89] used the ReliefF algorithm to assign different weights to features influenced by various factors. This fusion demonstrates strong robustness in data with multiple reproductive periods, multiple perspectives, multiple resolutions, and multiple lighting conditions. Qiu et al. [58] proposed a novel color feature GB to highlight the diseased parts of wheat ears in grayscale images, providing strong support for accurately detecting the diseased areas of different varieties of wheat (awned and awnless wheat with different resistance to FHB).

**Figure 4:**
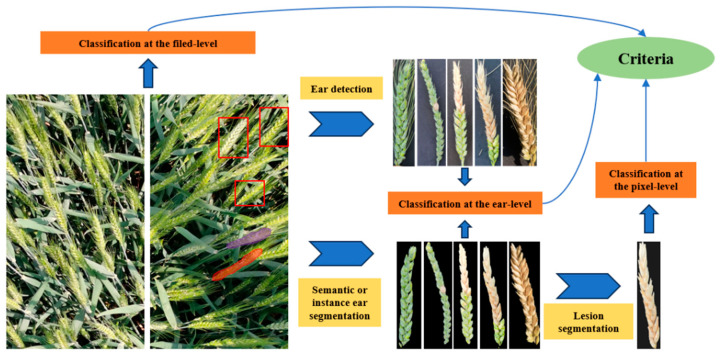
Schematic diagram of wheat ear FHB detection at different levels on the medium scale.

**Technology roadmap:** The wheat FHB detection based on RGB images at the medium scale often involves single or combinations of multiple tasks such as recognition, detection, classification, segmentation, and counting. The detection task in this section usually revolves around the severity rating of the target plot. Researchers can choose different technical routes based on different data types or research requirements. As shown in Figure 4, according to the modes of data analysis, it can be roughly divided into field-, ear-, and pixel-level. Firstly, wheat ear FHB detection at the field level directly predicts the severity rating of the corresponding plot using in-situ RGB images. Rößle et al. [88] introduced a dataset consisting of 3000 images of small-scale wheat fields and modeled based on the severity ratings of two independent agronomists. This method does not require image annotation based on pixels or individual wheat ears and can directly perform high-throughput detection on large fields. However, due to the complexity of the features of the complete plot, there remains much room for improvement in the accuracy of this method. Secondly, wheat ear FHB detection at ear level directly classifies the severity of FHB severities using a classification network based on segmented or detected wheat ears. Mao et al. [31] proposed a lightweight model with an average accuracy of up to 99.23% based on individual wheat ears with disturb of various complex backgrounds inside and outside. However, compared with other technical roadmaps, the analysis at the ear level makes more technical demands of data acquisition or preprocessing. The final and most precise technical roadmap, detection at pixel level, is a route that generally segments disease spots on the detected and segmented images of wheat ears without background and determines the severity of individual wheat ear diseases by calculating the number of pixels in the disease spots and whole ears. Su et al. [85,87] proposed cascaded segmentation networks to sequentially separate wheat ears in fields and disease spots on ears utilizing the same segmentation model. Zhang et al. [17] developed a complete method for the automatic detection of FHB severity in wheat fields by combining the Yolo detection network with twice unsupervised segmentation and counting. Although this technology route requires more manual annotation costs, its accuracy and interpretability are significantly superior to other roadmaps. 

**Application:** Due to the abundance of data and data types, as well as the strong learning ability of deep learning algorithms, many studies have achieved satisfactory results in the detection of wheat ears in natural conditions and demonstrated full applicability. Gao et al. [91] utilized only 3.64 M parameters and 4.77 G floating-point operations (FLOPs) to achieve 97.15% mAP, which can effectively meet the real-time, efficiency, and accuracy requirements of mobile port porting. Mao et al. [31] designed a mini program on a mobile device with an accuracy rate of up to 99.23% for single spike severity determination. The cluster spike severity monitoring model based on a dual-segmentation network constructed by [87] only requires a high-quality digital camera and a vehicle that can adapt to the field to be applied. The 360° phenotype robot designed by [86] can automatically capture images of wheat in all directions while detecting and segmenting diseased spikes. In addition, due to the extremely high shooting freedom of this device, the location of FHB can be detected at an earlier stage. The devices equipped with these applications have the characteristics of easy access, high reusability, and high generalization application value, while the applications themselves also have the characteristics of high usability, non-destructive, fast and accurate.

#### 4.3.2. Detection Based on Spectral Imaging

Spectral imaging can obtain deeper information from wheat than RGB imaging [92]. In addition to severity rating at the late growth stages, spectral imaging also undertakes the key task of wheat FHB early detection. Earlier and faster detection of FHB is of great significance for effectively isolating wheat-containing toxins and reducing losses.

**Data acquisition:** The outdoor environment has significantly impacted spectral imaging, and previous conventional methods have mainly focused on laboratory conditions [93]. Currently, researchers are increasingly choosing to use black cloth as the background when imaging outside [94] or directly performing in-situ detection on wheat [95]. Models based on complex imaging environments can better adapt to the usage scenarios of methods. However, diverse sampling methods can reduce the requirements for image acquisition and obtain more directional information on diseased wheat ears. [96] and Zhang et al. [97] captured wheat ears from the front and back to obtain spectral information on them with possible differences in infection levels on each side. Mustafa et al. [98] captured the top, middle, and bottom of each wheat spike from both sides to obtain the chlorophyll content of each partition. Multi-directional sampling not only provides more comprehensive and accurate spectral information but also reduces the influence of natural factors such as atmosphere, lighting, and shadows through averaging processing, improving the uniformity of surface reflection of objects. They all will make specific spectral features more obvious and detectable.

**Data processing:** Traditional feature extraction and selection methods are not flexible enough to be used in more complex imaging scenarios. Although various traditional vegetation indices, including NDVI and SIPI, have been proven to have a high correlation with the severity of FHB [99], the classification results have not been satisfactory in practice [93]. Therefore, as shown in Table 2, researchers designed a series of specific FHB detection indices based on the physiological and biochemical changes of diseased wheat. In corresponding studies, these indices have demonstrated stronger FHB severity grading ability compared to traditional indices. These indices often provide a more sensitive response to biochemical changes induced by FHB, making them easier to capture with imaging systems. For example, by analyzing the performance of WSFI_2_ in hyperspectral data, a component of the specificity index WSFI in Table 2 [100] demonstrated the invasion of FHB first at pigments and second cause structure damage. They also revealed that the hemi-biological behavior of Fusarium can lead to strong signals in wheat ear spectra at 865 nm, even at very low disease severity.

Moreover, data fusion breaks the shackles of traditional feature selection methods and provides more comprehensive considerations for decision-making under fine FHB severity grading indicators. In the heterologous data fusion, Mustafa et al. [98] utilized the Machine Learning Sequential Floating Forward Selection (ML-SFFS) algorithm to classify the severity of FHB infection into nine levels based on multimodal data fused with HSI, CFI, and HTP; Mahlein et al. [101] refined the classification criteria to ten levels by fusing data from three imaging techniques: IRT, CFI, and HSI. Both of them achieved an average accuracy of over 85%. Among the above technologies, HSI can provide triple information including space, radiation, and spectrum, IRT can provide temperature information of the primary infection site, CFI can provide parameters such as maximum fluorescence efficiency to accurately evaluate the photosynthetic efficiency and health status of wheat ears, and HTP can provide technical support for monitoring plant growth from multiple time points. In homologous data fusion, H Huang et al. [32] integrated traditional spectral features, color features, and texture features, achieving a five-level severity classification with an accuracy rate of 92%. Mustafa et al. [100] achieved a nine-level classification with an accuracy of over 80% under multiple spatiotemporal conditions by fusing wavelet features and texture features.

**Table 2:** Specific FHB index based on hyperspectral imaging.

Nomination	Scene	Index Formulation	Reference
FCI	Lab	FCI = 0.25*2(R668 − R417) − R539	[102]
FDI	Lab	FDI = (Rλ1 − Rλ2)/(Rλ1 + Rλ2)	[60]
WFSI and WFTI	Lab	WFSI = (W1 − W2)/(W1 + W2) WFTI = (T1 − T2)/(T1 + T2)	[100]
WSI	Field (black background)	WSI = (SD_450–488_ − SD_500–540_)/ (SD_450–488_ + SD_500–540_)	[103]
WFCI1 and WFCI2	Field (in situ)	WFSI = (R401 − R840)/(R401+ R840) WFTI = (R460 − R786)/(R460 + R786)	[104]
WFItwo and WFIthree	Field (in situ)	WFItwo = (R687 − R760)/(R687 + R760) WFIthree = (R760 − R687)/(R687 + R659)	[105]

Rn: Spectral bands at n nm; Wi: wavelet feature; Tj: texture feature.

The wheat FHB detection based on spectral data often uses one-dimensional spectral data and establishes models after major steps such as preprocessing, feature extraction, and feature selection. During such a process, more accurate results often mean more complex algorithm design. Deep learning methods alleviate the problem of complex manual model design, but relying solely on them for one-dimensional data analysis is not comprehensive enough. Jin et al. [106] applied deep neural network classification algorithms to hyperspectral pixels, capturing the intrinsic features of hyperspectral images from two-dimensional data. Hamila et al. [55] obtained 3D point cloud data consisting of RGB and NIR color channels using a multispectral 3D scanner, obtaining more detailed representations of object edges, surfaces, and textures than standard 2D images. Aravind et al. [20] explored four different types of RGB image conversion schemes based on HSI, resulting in spectral (line and bar) graphs, compressed spectral line graphs, and 2D-generated band images. In addition, from their visualization analysis of the four types of images, it can be seen that the spectral region with higher contribution in the spectral line graph and 2D generated image has a high correlation with previous one-dimensional data research results. Compared with one-dimensional data processing, the processing of multidimensional data is not more complex with the assistance of various previous feature processing and decision algorithms but more diverse. Both feature fusion and decision fusion make the final model learn richer content and obtain more stable results [32,107].

**Task orientation:** The main direction of wheat FHB automatic detection based on spectral data at the mesoscale is disease severity grading or asymptomatic detection. Most of the above studies focused on disease severity grading and achieved reliable performances. Asymptomatic detection is the most significant and difficult part of early automatic FHB detection. Due to the varying relative importance of each input indicator depending on the severity of the disease [108,109], many research results based on symptomatic wheat are difficult to directly apply to asymptomatic detection. Mustafa et al. [98] successfully amplified the internal weak infection characteristics of asymptomatic wheat by utilizing CFI and combining sensitive features, achieving an average classification accuracy of 87.04%. The appearance of asymptomatic wheat infected with FHB and the healthy one is highly similar, so the acquisition of asymptomatic samples and high errors in manual annotation have always been the difficulties of the detection task. Jin et al. [110] proposed a three-stage neural architecture search technology based on transfer learning, which alleviated the issue of high acquisition costs for asymptomatic wheat data by zoning planting and inoculating spore bacterial solution while achieving an accuracy of 90.42% in three-classifications task (health, symptomatic, and asymmetric). Even if it remains impossible to fully determine whether some asymptomatic wheat ears have been mixed into the data with the true identity of healthy wheat ears, it will result in dirty data. Integrating data sources or conducting multi-fold cross validation under a unified data source to reduce the frequency and degree of impact caused by data anomalies may tackle this issue.

**Application:** Instruments or sensors created utilizing less wavelength information or spectral features can reduce manufacturing costs and data processing time. The several wheat FHB specificity indices in Table 2 can reduce stray light or interference signals unrelated to the required signal and improve the accuracy of the spectrometer. High specificity also means that the instrument can better distinguish different signal sources, making it easier for operators to set up and calibrate the spectrometer, thereby simplifying the instrument’s usage process. In addition, multiple methods based on wheat canopy [104] and wheat ear side [96] images have also demonstrated stability and accuracy that can be combined with multi-angle UAV technology. Such high-throughput spectral detection has also been proven to be fast, efficient, non-destructive, and more cost-effective in other crop detection [111,112].

### 4.4. Challenge on Ears Detection

#### 4.4.1. Part of RGB Imaging

Barbedo [22] detailedly discussed the difficulties faced by crop disease detection based on RGB images at the medium scale. The problem of unclear symptom boundaries related to wheat FHB detection can now be well solved through precise unsupervised segmentation networks [35]. However, the problem of overestimating the severity caused by the high similarity in color among wheat awns, healthy wheat ears, and diseased areas at the maturity stage is still not well solved by relying on RGB imaging. Moreover, the complex issue of background separation proposed by [22], namely the wheat ear segmentation problem in FHB detection, can still be tackled by semantic or instance segmentation with complex annotations or unsupervised segmentation models with poor accuracy. The errors of a single front-end task (ear segmentation) often sustainedly impact subsequent tasks and significantly interfere with the final decision. In addition, on the data processing side, some other challenges need to be faced, and these mainly focus on eliminating the negative impact of multivariate data, such as random noise, data imbalance, and so on. On the application side, model lightweight technologies have been widely applied at this imaging scale, and most models have been verified to own the ability to be mounted on edge devices. However, the various impacts of different edge devices on the models have been proven to be complex [113,114], and the impact on wheat FHB detection is still urgent to be explored.

#### 4.4.2. Part of Spectral Imaging

As the experiments of wheat FHB detection based on spectral imaging at the medium scale gradually shift to field trials, the removal of complex backgrounds is a challenge that must be tackled in the next stage of research. Almoujahed et al. [95] successfully customized wheat ear segmentation in hypercubes using the superpixel algorithm on NDVI images, but the time cost of data smoothing far exceeded the range that real-time detection tasks can withstand. This also exposes the problem of slow automatic data processing due to the large amount of information in spectral imaging. Huang et al. [34] demonstrated that in-situ detection, wheat samples with leaves have a greater impact on the model compared to samples without leaves. Accordingly, the segmentation of wheat leaves also has become an essential part of in-situ detection. Moreover, from the perspective of experimental environment adaptation, the noise caused by various uncertain conditions of lighting conditions or spectral equipment in field trials also needs to be tackled seriously. From the perspective of model construction and variable management, variables such as the different sensitive wavelengths of different FHB-resistant wheat seeds [99] and the varying FHB severities during the various growth stages [115] both require more sophisticated feature selection and model design to control and utilize.

## 5. Detection of Wheat FHB at Submacroscopic Scale

### 5.1. Overview of Previous Research

Currently, the tasks at the submacroscopic scale mainly revolve around drones, aiming to achieve large-scale detection of FHB and guide precise pesticide spraying. Unmanned aerial vehicles (UAVs) remote sensing images possess spatial resolution as high as centimeter-level, which can meet the requirements of rapid and accurate detection and real-time monitoring of crop diseases in large-scale planting areas as much as possible [116]. Moreover, compared with other crop diseases, wheat FHB is more suitable for UAV remote sensing monitoring [117]. FHB first infects the top of wheat ears and causes the appearance of a pink mold layer; therefore, after the diseased parts wither and brown, the spikelets above the affected area will become chalky white. Moreover, the top white spikelets are not obstructed by wheat awns or leaves, allowing drones to directly identify the diseased parts of wheat from above [118].

In the application of RGB imaging at a submacroscopic scale, in order to obtain clear and abundant wheat ears images, the flying altitude of drones is generally around a few meters. The data analysis approaches mainly rely on deep learning models, and the annotated objects are generally whitening or wrinkled spots, pink molds, or black granular lesions on spikes. These detection targets often have trouble with unclear features and inconsistent scales. Bao et al. [37] constructed an adaptive spatial feature fusion network based on contrast-enhanced wheat RGB images. This method not only improved the detection accuracy but also tackled the above-mentioned issues caused by the small size of the infected areas in images. In addition, the wind field generated by the low-altitude flight of drones can lead to inconsistency in the direction and scale of wheat ears, indirectly resulting in imbalanced data distribution in the training data. To address these two issues, Bao et al. [38] validated the impact of model accuracy and addressed them using random affine rotation enhancement and histogram distribution statistical methods. Furthermore, they proposed a cubic power stretching illumination processing algorithm to solve the problem of image overexposure, and the lightweight detection model proposed by [117] could provide strong technical support for the practicalization of drones on this spatial scale.

The research on spectroscopic techniques at a submacroscopic scale is often based on images captured at altitudes of tens or even hundreds of meters in order to obtain sufficient spectral data of scale. It is extremely difficult for only visible light information to be effective at this height. For target areas of the same size, the higher the flying altitude of the drone when obtaining the images, the lower the spatial resolution of them. Better spatial resolution can ensure image clarity while accelerating image processing efficiency. Zhu et al. [40] obtained wheat FHB data with six different spatial resolutions from a multispectral camera mounted on a drone. They determined the optimal spatial resolution by modeling vegetation indices and texture features extracted from spectral images. Texture, as one of the most commonly used features in this task [119], could be used to describe the brightness spatial distribution of adjacent pixels and reflect the spatial pattern of field-scale diseases. Xiao et al. [120] selected the optimal window size of the Gray Level Co-occurrence Matrix (GLCM), which is the most classic and universal method for extracting texture features. However, the changes in chlorophyll and water loss at the mature stage of wheat will greatly reduce the effects of spectral and texture features in modeling at the submacroscopic scale. In addition to these two features, color features have been proven to be the most effective means of identifying wheat FHB [121]. As the infection spreads, the diseased state of the various wheat organs and tissues usually transitions from green healthy to withered yellow or chalky white. Hence, the addition of color features enabled the model to capture more wheat with more severe disease conditions [25]. Generally, images captured at the submacroscopic scale cannot obtain sufficient internal information on wheat and often require more different types of information to assist the model establishment. As shown in Table 3, multi-source data fusion has assisted many studies in achieving reliable accuracy.

### 5.2. Challenge

Firstly, imaging at the submacroscopic scale often requires clear and windless weather with little variation in light-intensity conditions. Therefore, improving the accuracy of preprocessing techniques and reducing the impact on effective data to overcome complex natural factors remained a major challenge. Secondly, the flexibility and efficiency of drones have reduced the difficulty of data acquisition at the submacroscopic scale, accelerating the development of large-scale automatic detection of wheat FHB. However, drones are prone to causing spectral equipment vibration during flight, making the captured images unclear. Manual cropping is necessary to move contaminated image information [95], but reducing the labor-intensive level of this work and promoting its automation is more important. 

Furthermore, the model established based on RGB images obtained from drones not only needs parameter reduction to make the images lightweight but should consider the impact of differences between various edge devices on them. Finally, the uneven wheat varieties or growth periods in the planting area will also pose significant obstacles to the practical application of models based on spectral information, which relies on internal tissue information of wheat with FHB [99]. Therefore, reasonable planning of planting varieties and planting times is also very helpful for the automatic detection work. It should be further emphasized that overcoming these challenges is crucial for the large-scale promotion of automatic and accurate detection of wheat FHB.

## 6. Detection of Wheat FHB at Macroscopic Scale

### 6.1. Overview of Previous Research

**Figure 5:**
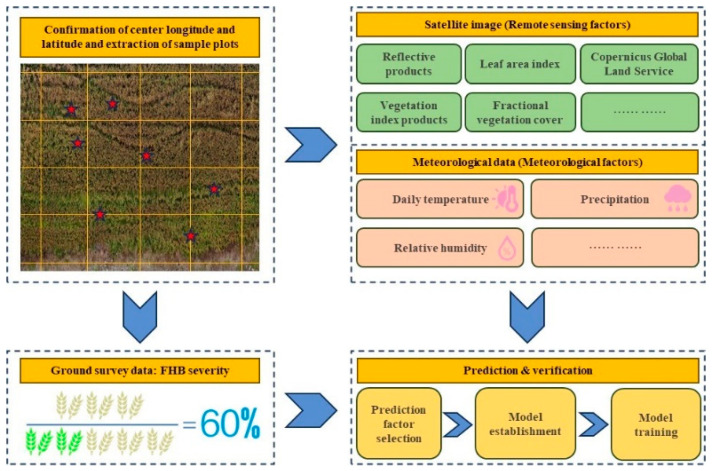
Basic workflow for predicting the severity of wheat FHB at macroscopic-scale imaging. The red pentagram in the upper left corner of the figure represents random sampling points, while the green wheat in the lower left corner represents healthy wheat and the yellow wheat represents diseased wheat.

The automatic detection of wheat FHB at a macroscopic scale mainly revolves around satellite remote sensing technology. As an economical and independent large-scale synchronous observation method, satellite remote sensing technology has developed into a better choice for crop disease detection based on macroscopic-scale imaging [122]. The researchers mainly predicted the growth stage of regional wheat fields, or predicted, simulated, detected, or mapped regional FHB situation with satellite products. The data these tasks obtained was not only massive but with more complicated structures. The basic workflow for predicting the severity of wheat FHB at the macroscopic scale is shown in Figure 5. Appropriate temperature, rainfall, and relative humidity are the main influences on the spread of wheat FHB, so the combination of meteorological and remote sensing factors is the critical link in the automatic detection of wheat FHB. Xiao et al. [42] accurately predicted the severity of FHB in two regions by combining remote sensing variables and weather variables, taking into account host and environmental conditions. In addition, they achieved higher accuracy during the heading and flowering stages by utilizing time-varying features for dynamic prediction. Meanwhile, [41] delved into the particularly important relative humidity, temperature, and phenological conditions in environmental and host conditions. They introduced a variable designed based on the three conditions above, which overall showed a high correlation with disease incidence and demonstrated an affinity between the predicted results and the conditions. Furthermore, considering the differential sensitivity of wheat to FHB at different growth stages and the complexity of satellite remote sensing data processing, the spatiotemporal generalization ability of detection methods is particularly important. Based on multi-growth stage data, Li et al. [26] captured the spatiotemporal changes of wheat FHB by combining multi-scale meteorological factors and time-varying remote sensing factors and achieved high-precision prediction of wheat FHB. However, for spectral data, we found that remote sensing has a stronger dependence on vegetation indices compared to ground-based remote sensing. However, common vegetation indices do not have specificity or dependence on disease, which makes it difficult for models to accurately capture the characteristics of a specific disease. Liu et al. [54] developed a novel wheat FHB-specific monitoring index based on simulated satellite multispectral data by analyzing the red edge (RE) spectral region and some basic vegetation indices. The addition of the RE band and the proposal of this index have improved the estimation accuracy of leaf area index, chlorophyll, and nitrogen content in wheat infected with FHB [122,123].

### 6.2. Challenge

From the perspective of data types, current research at the macroscopic scale is often based on satellite data corresponding to standardized farmland. The varieties, planting procedures, and human management measures of these experimental fields are relatively uniform. Practically, merely differences in wheat varieties can lead to differences in spectral characteristics [99]. This may be achieved by establishing a reference spectral library for different wheat varieties and matching and correcting the data during processing, but it must be a massive project that requires plenty of manual data collection. The diversity and differences in planting strategies among individual farmers can also pose a major challenge to existing models or specificity indices. For the diversity of individual growers, farmland can be divided into different types of areas or clusters, and suitable models can be established for each type of area or cluster. It can make the detection techniques focus more on its generalization ability in this type of region, thereby improving its accuracy. In terms of data composition, there remains room for improvement in the accuracy of most existing methods. Many researchers in other fields are attempting to introduce more variables, such as wind speed [124] and accumulated temperature [125], as input variables to provide more comprehensive information for the model. Simultaneously, this also puts higher demands on the preprocessing techniques used to reduce the computational complexity of the model. In addition, there remain many issues that need to be tackled in data processing, such as the spatial clustering changes of the SEIR model in crop disease simulation [126] and the impact of mixed pixels on phenological information extraction [42]. The introduction of spatial statistical techniques and mixed pixel unmixing techniques may optimize the original solution, but how to cleverly combine these techniques with the original image processing techniques remains a technical bottleneck that needs to be overcome in the future.

## 7. Future Perspectives

The yield loss caused by FHB and the contamination of wheat kernels with mycotoxins is becoming increasingly severe. This highlights the need for effective tools for real-time field detection and identification of FHB. With the booming development of automatic non-destructive wheat FHB detection, we believe that these detection technologies will gradually achieve full coverage of the entire growth period of wheat. At the breeding stage, automatic nondestructive detection for mesoscale FHB will reduce the cost of phenotypic analysis of wheat kernels and help breeders quickly and extensively screen out healthy wheat. Especially with the support of conveyor imaging systems, data collection, and quantitative and qualitative evaluation will be faster and more accurate [127]. At the early stage of pathogenicity, spectral imaging technology at various scales will help to achieve accurate FHB spore detection in the field, precise application of fungicides [128], and effective control of disease incidence rate. At the development stage of the FHB, smart mobile devices equipped with detection technologies will assist agricultural producers and management personnel in detecting the disease and getting a clearer understanding of the disease severity in a timely and autonomous manner. For example, a mini program that can be directly applied to determine the severity of single wheat spike FHB in the field [31], or a plot disease severity determination method that combine detection, twice segmentation, and counting [17]. A real-time FHB monitoring system at a larger scale will enhance the management of unmanned farms [129], disease control, and precision employing pesticides through drones or satellite monitoring systems. Selective harvesting of healthy wheat at the maturity stage, the period of FHB outbreaks, is an option to avoid infected spikes and reduce mycotoxin contamination [116]. At this moment, large-scale, high-throughput, non-destructive testing technology will support the reapers in quickly confirming the areas that need to be harvested and assessing yield loss in order to harvest as many healthy wheats as possible. The stability of this technology requires the method to be tested in real or convincing simulation environments [117,130] and achieve high-quality results.

The long-term development of automatic non-destructive FHB detection technology is undoubtedly the combination of the various automatic detection tasks. This requires the mature development of detection technologies at various imaging scales and the fusion of advantages within the same framework. The functions of real-time monitoring and data collection, big data analysis, disease monitoring, early warning, precise pesticide use and fertilization, automation, and remote control will all form an ideal automatic, non-destructive detection system. To achieve such a combination, reliable and efficient sensor technology, extensive data transmission and storage capabilities, ensuring data privacy and security, effective data sharing and collaboration mechanisms, and ensuring communication, network latency, and device compatibility will all need to be extensively considered. From this perspective, IoT technology is a promising tool for future wheat FHB automatic non-destructive detection systems [131]. Correspondingly, the integration of multi-source imaging technology may also be a potential development trend.

## 8. Final Considerations

FHB has brought significant economic losses and food security risks to global wheat production, especially under the impact of climate change in recent years. Through continuous exploration and research, many effective methods for FHB detection, monitoring, and severity classification have been accumulated. Professional visual and internal physiological structure analyses have also been significantly developed. This article reviewed the main work and challenges of automatic non-destructive detection technology for wheat FHB at four scales: microscopic, medium, submacroscopic, and macroscopic scales. The detection targets in the reviewed research included but were not limited to Fusarium spores, FDKs, monomeric wheat ears, small and medium-sized, and regional wheat field plots. For wheat FHB, research at the microscopic scale provides a control basis for controlling the source of the disease, and overcoming the complex and diverse microbial populations in farmland is a necessary path to achieve the application of technology in the field. The accessibility of detection techniques obtained at medium-scale research is of great significance for the popularization of technology, and higher quality generalization or specificity capabilities will pose challenges for future research. Research at the submacroscopic scale is rapidly advancing toward high-throughput, non-destructive detection with the support of UAV technology. Coordinating the relationship between planting management and the detection scope will be of great benefit to the implementation of technology. Research at the macroscopic scale provides a large-scale synchronous observation method for researchers to predict and plot FHB situations, and treating non-standard experimental fields as detection targets to enhance the generalization ability of the method will be the focus and difficulty of the next stage of work.

Overall, these technologies at various imaging scales have been proven to be effective tools for managing and controlling FHB, and their application almost covers the entire growth cycle of wheat. The actual impact on agriculture and food security is undoubtedly positive. These technologies provide fast, accurate, and non-destructive testing methods, improving the efficiency of disease monitoring and management and helping to ensure the quality and sustainable development of food production. However, in the process of promotion and application, it remains necessary to address issues such as technical cost and accuracy in order to further enhance the feasibility and practicality of the technology.

## Figures and Tables

**Table 1:** List of partial contributions according to ACDD based on multi-scale imaging.

Imaging Scale	Filming System	Detection Task	Evaluation Metric	Reference
Microscopic	Microscope with digital camera	Wheat FHB fungus detection	Accuracy 0.9857	[24]
	Electron microscope	Wheat FHB spore segmentation	F1 0.943; mIoU 0.925	[28]
	Micro-near-infrared spectrometer	Early FHB with asymptomatic grains prediction	mAP 0.88	[29]
	Hyperspectral camera	Healthy and diseased ears discrimination	Accuracy 0.99	[30]
Medium	Digital camera and mobile phone	Severity of wheat ears FHB identification	mAP 0.9923	[31]
	Spectrometer and digital CCD camera	Severity of wheat ears FHB Diagnosis	Accuracy 0.92	[32]
	NIR camera	Three classes of FHB severity discrimination	Sensitivity 0.994; Specificity 0.919	[33]
	Portable spectrometer	Wheat ears FHB identification	Accuracy and Kappa: leafy 0.65, 0.27; leafless 0.81, 0.63	[34]
	SLR camera	Severity of wheat ears FHB identification	Accuracy 0.925	[35]
	Benchtop hyperspectral imaging system	Analysis of damaged wheat kernels	mAP 0.97	[36]
Submacroscopic	UAV with RGB camera at 4 m	Wheat FHB ears detection	AP 0.808; Recall 0.743; Precision 0.779	[37]
	UAV with RGB sensor at 4 m	Wheat FHB ears detection	mAP 0.832; Recall 0.745; Precision 0.806	[38]
	UAV with multispectral camera at 60 m	Wheat FHB monitoring	Overall accuracy 0.98	[39]
	UAV with hyperspectral camera at 60 m	Wheat FHB detection	Accuracy 0.83	[25]
	UAV with multispectral camera at 20–110 m	Wheat FHB monitoring	R2 0.83; RMSE 3.35; RPD 2.72	[40]
Macroscopic	Satellite MODIS and Sentinel-2 and 3	Wheat FHB prediction	Overall accuracy 0.88 in April and 0.92 in May	[26]
	Satellite MODIS and Sentinel-2	Wheat FHB regional prediction	RMSE 0.131; Acc 0.860	[41]
	Satellite MODIS and Landsat-8	Wheat FHB severity prediction	mAP 0.8175	[42]

**Table 3:** Research on multi-source data fusion for automatic detection of wheat FHB.

Detection Task	Feature Type	Evaluation Metric	Reference
Severity monitoring	5VIs + 1TF + 1SB	AUC 1.0, SD 0.0 and Accuracy 0.98	[39]
Disease detection	10VIs + 3TIs	Accuracy 93.63% and F1-score 92.63%	[119]
Severity monitoring	5VIs + 9TFs	R2 0.83, RMSE 3.35 and RPD 2.72	[40]
Severity monitoring	3SFs + 3TFs + 2CFs	Accuracy 85%	[38]

(VI = Vegetation index, TF = Texture feature, TI = Texture index, SB = Spectral band, CF = Color feature).

## Data Availability

No new data were created or analyzed in this study. Data sharing is not applicable to this article.
